# Unrecognized psychiatric disorders among adult patients admitted into a general hospital in Maiduguri, Northeastern Nigeria

**DOI:** 10.11604/pamj.2014.19.197.4531

**Published:** 2014-10-24

**Authors:** Abdulaziz Mohammed, Jidda Mohammed Said, Musa Abba Wakil, Isa Bukar Rabbebe, Taiwo Sheikh, Samuel Agunbiade

**Affiliations:** 1 Department of Clinical Services, Federal Neuro-Psychiatric Hospital Kaduna, Kaduna state, Nigeria; 2 Department of Mental health, University of Maiduguri Teaching hospital, Maiduguri, Nigeria; 3 Department of clinical services, Federal Neuro-psychiatric Hospital Maiduguri, Borno state

**Keywords:** epression, anxiety disorders, prevalence, general practitioners, non-recognition, General hospital

## Abstract

**Introduction:**

Patients with unrecognized psychiatric disorders in general hospitals, suffer economic and psycho-social difficulties. This study aimed to determine (i) prevalence and pattern of psychiatric disorders, and (ii) prevalence of unrecognized psychiatric disorders among adult in-patients of a general hospital.

**Methods:**

In this two-stage, cross-sectional study, we used (i) General Health Questionnaire (GHQ) and Composite International Diagnostic Interview (CIDI) to assess the prevalence of psychiatric disorders, and (ii) Patient Encounter Form to determine unrecognized psychiatric disorders, among patients admitted into a general hospital.

**Results:**

Of the 283 respondents, 174 (61.5%) had GHQ scores of ≤ 4. Eighty seven respondents (31%) had psychiatric disorders of which 85 (98%) were not recognized. The frequency of Depression and Anxiety disorders were 61.5% and 26.2% respectively. Unmarried (2.3, 1.2-4.3; p < 0.00), females (2.1, 1.1-4.05; p = 0.01) and patients with “unexplained symptoms” (≤ = 8.4, p< 0.00, df = 1) were more likely to have diagnosis of depression and anxiety disorder.

**Conclusion:**

We conclude that one-third of the patients in the general hospital, had co-morbid psychiatric diagnoses, mostly unrecognized by their physicians. Unmarried, females and respondents with unexplained symptoms were associated with depression and anxiety disorders. We recommend the posting of psychiatric trainees to general hospitals, and training of general practitioners on the use of simple depression and anxiety screening instruments.

## Introduction

The prevalence of psychiatric disorders among patients admitted into general hospitals compared to that of community [1], is higher, it ranges between 16 to 61 percent [2–4]. Depression and anxiety disorders were the most common psychiatric disorders reported in general hospitals [5]. Patients with unrecognized or misdiagnosed psychiatric disorders in general hospitals, fail to receive appropriate treatment [6, 7], and this has economic and psychosocial implications (increased cost of health care, increased length of hospital stay, increased rates of readmissions, decreased quality of life), and increase mortality [8]. Non-recognition of psychiatric disorders by general practitioners in many developing countries may lead to poor referral of patients to psychiatric hospitals, and patients’ poor access to mental health care services, especially in places where there is shortage of mental health specialists. The present study aimed to determine (i) prevalence and pattern of psychiatric disorders, and (ii) prevalence of unrecognized psychiatric disorders among adult in-patients of a general hospital.

## Methods

### Study setting

We conducted this study at the General Hospital, Maiduguri, in North-eastern Nigeria. The hospital receives referral from all the Primary Health Care (PHC) Centers and cottage hospitals in the state.

### Study design

This is a cross-sectional hospital-based study of male and female patients aged ≥ 18 years, admitted into the medical, surgical and gynaecological wards of the State General Hospital from 14th to 28th April, 2008. We excluded unconscious patients and those who refused consent.

### Sample size determination

We calculated a minimum sample size of 136, using the Leslie and Kish [9] formula for estimating sample size:

n= (Zα2pq)/ d^2^

Where: n = minimum sample size; Zα = set at 5% significant level = 1.96; p = estimate of prevalence of psychiatric morbidity among patients admitted to medical and surgical wards in a Nigerian study [10] = 30% = 0.3; d = level of precision (5%); q = 1- p.

Substituting in the above formula, n= (1.96^2^ x 0.30 x 0.70) / 0.05^2^ = 323

Since, the total in-patients population of the hospital is <10,000, the sample size was corrected using the formula:

nf= n/(1+(n)/N)

Where: nf = the desired sample size for a population < 10,000; n = the desired sample size for a population >10,000; N = the estimate of the total in-patients at the medical, surgical and gynaecological wards of the hospital = 165.

Substituting in the above formula,

nf = 323/(1+(323)/165), nf = 109

To compensate for possible non-response, we adjusted the calculated sample size, using the formula below: ns = nf/ar. Where: ns = the compensated sample size; nf = the calculated sample size (109) ar = anticipated response rate, set at 80%. (0.8).

Thus, ns = 109/0.8 = 136

To allow for test of associations, we increased the sample size to 300. Therefore a sample size of 300 was used for the study.

### Sampling Technique

We used systematic sampling technique to select samples for the study and divided the sample size of 300 between the wards (using proportional allocation method based on the ratio of their bed spaces). Thus, the medical and surgical wards with a bed space of 60 each were allocated 110 participants each, while the gynecological ward with a bed space of 45 was allocated 80 participants. In each ward, we selected every third patient admitted until the required sample size was achieved.

### Study Instruments

We used a pre-designed Socio-Demographic Questionnaire, the General Health Questionnaire (GHQ-28), Composite International Diagnostic Interview (CIDI), and the World Health Organization (WHO) Patient Encounter Form for data collection. The pre-designed socio-demographic questionnaire recorded information on participants’ bed number, ward, age, sex, occupations, and marital and educational status. We used General Health Questionnaire (GHQ-28) to screen the respondents for psychiatric disorders. It is a self-administered screening instrument that detects psychiatric symptoms in patients regardless of their diagnosis [11]. The Likert method for scoring GHQ was used, and set the cut off score at ≥ 4. GHQ-28 has been validated for use in Nigeria [12]. Staff of the Department of Languages and Linguistics, University of Maiduguri translated the GHQ-28 into Hausa language, using the back translation method, for respondents that do not understand English language. Both the English version and the Hausa translation were used in the study. Patients that scored ≥ 4 were recruited for the second stage interview. They were administered the Composite International Diagnostic Interview (CIDI) to generate a specific diagnosis. CIDI is a structured clinical interview instrument, derived from the National Institute of Mental Health (NIMH) Diagnostic Interview Schedule (DIS) and the Present State Examination (PSE). It was designed for cross-cultural epidemiological research of mental disorders. It generates both the International Classification of Diseases (ICD-10) and Diagnostic and Statistical Manual of Mental disorders (DSM- IV) diagnoses, it has good reliability and validity [13]. For this study, the modules of CIDI for the diagnoses of depressive disorders, anxiety disorders (Phobias, panic anxiety disorders, and generalized anxiety disorder), somatoform disorders and substance use disorders were used. The aim is to identify conditions that are known to be common in this setting [14]. A Hausa version of the CIDI developed by back translation method for a previous survey, [14] was used for participants who speak only Hausa. We adapted the patient encounter form (from the World Health Organisation collaborative study of psychological problems in general health care) to measure the level of psychological illness recognised by the general practitioner [15]. The form has sections for classification of presenting symptoms, overall rating of the patient's health, diagnosis and severity of the physical and psychological disorder and treatment offered for the psychological symptoms. The attending medical doctor was requested to fill the form.

### Data collection and procedure

We recruited two resident doctors with experience on data collection, and who speak both English and Hausa language fluently. They were trained on the use of the study questionnaires and interview techniques for this study. The duration for data collection was five weeks and each interview lasted 30 minutes.

### Ethical consideration

Research and Ethics Committee of Federal Neuro-psychiatric hospital, Maiduguri approved the study protocol, and we obtained permission for the study from General Hospital, Maiduguri. Patients gave their written informed consent and confidentiality of the patients was ensured. We gave medical advice to participants with psychiatric distress and referred those with serious distress to a psychiatric hospital for expert care.

### Data Entry and Analysis

We used SPSS version 13 to analyze the data and descriptive statistics to report frequencies, proportions and tables. Categorical variables were analyzed using Chi square (χ^2^) test, level of statistical significant set at p < 0.05, at 95% confidence interval.

## Results

Of the 300 participants recruited for the study, 283 (94%) completed the study. A total of 17 participants failed to complete the study: 14 were discharged early, 1 died and 2 with severe illnesses. The socio-demographic characteristics of those who failed to complete the study were similar to those who completed the study. The mean age of the participants was 37 years (SD 16, minimum 18, maximum 85). One hundred and seventy-four (61.5%) participants had GHQ scores of ≥ 4 (GHQ positive), 87 (31%) had CIDI diagnoses of psychiatric disorders (Table 1). Eighty five (98%) of the CIDI positive participants had unrecognized psychiatric disorders. The most frequent psychiatric disorders among the patients were depression (61.5%) and anxiety disorders (26.2%). The attending physicians treated the patients with unrecognized psychiatric disorders with nighttime sedation, vitamins/tonic and analgesic, (Table 1).

**Table 1:** Prevalence and pattern of psychiatric disorders among patients admitted into the medical, surgical and gynecological wards of State Specialist Hospital, Maiduguri, Nigeria-2009

Variable	n	%
GHQ* score (N= 283)		
≥ 4	174	61.5
< 4	109	38.5
CIDI** diagnosis (N= 283)		
Yes	87	31.0
No	196	69.0
Disorders (N = 126)***		
Depression	78	61.9
Anxiety disorders	33	26.2
Somatoform disorders	11	8.7
Substance use disorder	4	3.2
Recognition of psychiatric disorders by physician (n = 87)		
No	85	98.0
Yes	2	2.0
Treatment for psychological symptoms (n = 7)		
Vitamins/tonics	3	43.0
Night time sedation with hypnotics	2	28.5
Analgesics	2	28.5

* GHQ: General Health Questionnaire

** CIDI: Composite International Diagnostic Interview

*** N > 87 due to presence of co-morbid disorders

Patients with “unexplained somatic symptoms” were 16 times more likely to be GHQ positive (16.0, 2.2-300.0; ρ< 0.00) (Table 2). Unmarried (3.0, 1.5-6.1; ρ< 0.00), no formal education (2.1, 1.2-3.8; ρ< 0.01), and severely ill patients (3.6, 1.5-8.7; ρ < 0.00) were more likely to be GHQ positive (Table 2). Female (2.1, 1.1-4.05; ρ= 0.01) or unmarried (2.3, 1.2-4.3; ρ< 0.00) patients were 2 times more likely to have depression. “Unexplained symptoms” were also associated with depression (ρ= 8.4, ρ < 0.00, df = 1) (Table 3). Unmarried (5.6, 1.8-17.5; ρ < 0.00), female (3.3, 1.2-10.2; ρ= 0.01) patients and presence of “unexplained symptoms” (5.1, 1.0-9.5; ρ= 0.02) were associated with anxiety disorder (Table 4).

**Table 2:** Factors associated with positive GHQ score of ≥ 4 among patients admitted into the medical, surgical and gynecological ward of state specialist hospital Maiduguri, Nigeria– 2009 (N= 283)

Characteristics	GHQ (+) (%)		GHQ (-) (%)		OR (95% CI)		ρ-value
**Presenting symptoms**					16.0	(2.2-300.0)	< 0.00
Unexplained	22	8.0	1	0.0			
Physical/psychological	152	54.0	108	38.0			
Total	174	100	109	100			
**Rating of illness (n = 272)**					3.6	(1.5-8.7)	<0.00
Severe	37	14.0	8	3.0			
Mild to moderate	128	47.0	99	36.0			
Total	165	100	107	100			
**Married**					3.0	(1.5- 6.1)	< 0.00
No	50	18.0	13	4.0			
Yes	124	44.0	96	34.0			
Total	174	100	109	100			
**Education**					2.1	(1.2- 3.8)	0.01
No	62	22.0	23	8.0			
Yes	112	40.0	86	30.0			
Total	174	100	109	100			
**Sex**					1.6	(0.94- 2.73)	0.06
Female	121	43.0	64	22.0			
Male	53	19.0	45	16.0			
Total	174	100	109	100			
**Occupation**					1.4	(0.8- 2.3)	0.2
Unemployed	85	30.0	45	16.0			
Employed	89	31.0	64	23.0			
Total	174	100	109	100			
**Ward**					1.2	(0.7-2.1)	0.4
Medical	69	25.0	38	13.0			
*Surgical/Gynaecology	105	37.0	71	25.0			
Total	174	100	109	100			

**Table 3:** Factors associated with a diagnosis of Depression among patients admitted into the medical, surgical and gynecological wards of State Specialist Hospital Maiduguri, Nigeria – 2009 (N= 283)

Characteristics	Depression (%) N (%)		No depression N (%)		OR (95% CI)		ρ-value
Presenting symptoms*					χ = 8.4**	< 0.00	
Unexplained	8	5.0	0	0.0			
Physical/psychological	70	45.0	78	50.0			
Total	78	100	78	100			
**Married**					2.3	(1.2- 4.3)	< 0.00
No	26	9.0	37	13.0			
Yes	52	19.0	168	59.0			
Total	78	100	205	100			
**Sex**					2.1	(1.1- 4.05)	0.01
Female	125	44.0	60	21.0			
Male	80	28.0	18	7.0			
Total	78	100	205	100			
**Education**					1.1	(0.7-1.6)	0.7
No	25	9.0	60	21.0			
Yes	53	19.0	145	51.0			
Total	78	100	205	100			
Occupation*					1.1	(0.7-2.0)	0.6
Unemployed	37	13.0	93	34.0			
Employed	38	14.0	109	39.0			
Total	75	100	202	100			
**Ward admitted**					1.3	(0.7-2.3)	0.3
Medical	33	12.0	74	26.0			
Surgical/Gynecological	45	16.0	131	46.0			
Total	78	100	205	100			
Rating of illness*					1.5	(0.7-3.0)	0.3
Severe	16	6.0	29	10.0			
Mild to moderate	62	23.0	165	61.0			
Total	78	100	194	100			

* N< than 283

** **χ:** Chi square test

**Table 4:** Factors associated with a diagnosis of Anxiety disorder among patients admitted into the medical, surgical and gynecological ward of State Specialist Hospital Maiduguri, Nigeria- 2009 (N= 283)

Characteristics	Anxiety disorder (+)		Anxiety disorder (-)				
	N	(%)	N	(%)	OR (95% CI)		ρ-value
**Married**					5.6	(1.8-17.5)	< 0.00
No	7	25.0	12	5.6			
Yes	21	75.0	201	94.4			
Total	28	100	213	100			
**Sex**					3.3	(1.2-10.2)	0.01
Female	28	84.9	157	62.8			
Male	5	15.1	93	37.2			
Total	33	100	250	100			
**Presenting symptoms**					5.1	(1.0-9.5)	0.02
Unexplained	6	18.0	17 233	17.0			
Physical/psychological	27	82.0	250	93.0			
Total	33	100		100			
**Rating of illness**					1.1	(0.0-5.6)	0.91
Severe	2	11.0	19 167	10.2			
Mild to moderate	16	89.0	186	89.8			
Total	18	100		100			
**Education**					0.85	(0.40-1.9)	0.66
No	17	51.5	139	55.6			
Yes	16	48.5	111	44.4			
Total	33	100	250	100			
Occupation					1.6	(0.7-3.6)	0.2
**Unemployed**	19	57.6	111	45.5			
Employed	14	42.4	133	54.5			
Total	33	100	244	100			
**Ward admitted**					0.9	(0.4-2.1)	0.9
Medical	12	36.4	95	38.0			
Surgical/Gynecological	21	63.6	155	62.0			
Total	33	100	250	100			

## Discussion

In this study, we found that a third of in-patients with physical illnesses at the State General Hospital Maiduguri also had a co-morbid psychiatric diagnosis, and 98% of the psychiatric disorders were unrecognized by the attending physicians. Unmarried, female patients with unexplained symptoms were associated with diagnoses of depression or anxiety disorder. The prevalence of 31% for psychiatric disorders among the patients in this study, is similar to the 30% reported by Abiodun [10] in medical and surgical wards of a general hospital. Another study,[16] of general hospital patients in a similar setting in Nigeria reported a higher prevalence of 45%. The study recruited elderly subjects (60 years and above) and thus included disorders more prevalent among elderly patients such as dementia which may have accounted for the difference in the prevalence. The most common psychiatric disorders found among patients in the general hospital in the present study were depression and anxiety disorders. This finding is similar to that of earlier studies among patients admitted into general hospitals [17, 18]. Psychotic disorders could potentially be easily recognized as requiring either psychiatric or alternative medicine care by relatives of patients, thereby presenting to either a psychiatric hospital or a traditional medicine center as opposed to depressive or anxiety disorders that may be mistaken as part of the physical condition and therefore patients encouraged to present to the general hospital.. In our sample, substance use disorder had the lowest prevalence of 1.4%. A possible explanation for the low prevalence is that patients refused to provide information on their substance use behavior because of cultural and religious reasons. Some patients may not consider substance use as worthy of mentioning because they fail to see it as a disorder. Binitie, [17] reported that Nigerians do not regard substance use (Alcoholism) as evidence of mental illness.

The rate of non-recognition of psychiatric disorders by medical officers in this study was 97.7%. This rate is higher compared to that of previous studies [19–21]. Knights, [21] found that doctors compared to nurses had lower rate of non-recognition of psychiatric disorders (30% vs.70%). Uwakwe, [16] reported a non-recognition rate of 97.2%, in Nigeria, similar to the rate found in this study. The very high rate of non-recognition found in this study is because of non-existence of consultation-liaison psychiatric practice in the hospital. Studies, [22] however, showed that religion, culture and the African worldview affect the diagnosis of depression among African population. We found association between unmarried, female patients and presence of “ unexplained symptoms” with depression and anxiety disorders. The medical officers considered depressive or anxiety symptoms of their patients as “unexplained symptoms” and thus failed to identify the psychiatric disorders. The non recognition could have been worsen by somatic symptoms, a common feature of depression in Nigeria[23]. This study was conducted in one hospital only and therefore limits the generalization to other hospitals but we are confident that the findings may apply to other general hospitals in the northeastern zone of Nigeria that share similar work force and patient background with the General Hospital Maiduguri

## Conclusion

We conclude that a third of patients presenting to general hospital Maiduguri for a physical condition had co-morbid psychiatric diagnosis, with most of the psychiatric disorders unrecognized by the attending physician. Depression and anxiety disorders were the most common psychiatric diagnoses among the patients. Unmarried, females and patients with unexplained symptoms were associated with depression and anxiety disorders. We recommend the posting of psychiatric trainees to general hospitals as part of their community psychiatric posting to practice consultation liaison psychiatry, and training for medical officers on how to use simple depression and anxiety screening instruments. Educating medical officers on the differential diagnosis of depression and anxiety disorders, especially in unmarried, female and patients with unexplained symptoms is necessary.
